# Prevalence, Wellbeing, and Symptoms of Dysmenorrhea among University Nursing Students in Greece

**DOI:** 10.3390/diseases7010005

**Published:** 2019-01-08

**Authors:** Eugenia Vlachou, Dimitra Anna Owens, Maria Lavdaniti, John Kalemikerakis, Eleni Evagelou, Nikoletta Margari, Georgia Fasoi, Eftychia Evangelidou, Ourania Govina, Athanasios N. Tsartsalis

**Affiliations:** 1 Department of Nursing, University of West Attica, 12243 Athens, Greece; ikalemik@uniwa.gr (J.K.); elevagel@uniwa.gr (E.E.); nmargari@teiath.gr (N.M.); gfasoi@uniwa.gr (G.F.); ugovina@uniwa.gr (O.G.); 2 Psychologist, MSc, 19009 Athens, Greece; demikitty@hotmail.com; 3 Department of Nursing, Alexander Technological Educational Institute, 57400 Thessaloniki, Greece; maria_lavdaniti@yahoo.gr; 4 Department of Infection Control, G.N.N. Ionias “Konstantopouleio—Patision” hospital, 14233 Athens, Greece; efievaggelidou@yahoo.gr; 5 Department of Endocrinology—Diabetes and Metabolism, Naval Hospital of Athens, 11521 Athens, Greece; ttsartsal@yahoo.gr

**Keywords:** dysmenorrhea, prevalence, pain intensity, menstruation, wellbeing, associated factors, nursing students, Greece

## Abstract

Dysmenorrhea (pain during menstruation) is one of the most common medical conditions among women of reproductive age. Dysmenorrhea has been studied around the world but not yet in Greece. The aim of the present study was to investigate the prevalence, characteristics, and impact of dysmenorrhea on the wellbeing (exercising, and social and academic functioning) among nursing students in Greece. A cross-sectional study of 637 nursing students was conducted by administering a questionnaire at a university in Athens. The prevalence of dysmenorrhea was 89.2% and the rate of severe intensity was 52.5%. Factors that were associated with severe dysmenorrhea were family history (*p* = 0.02), early menarche (*p* = 0.05) and menstruation duration (*p* = 0.05). Women with moderate and severe pain reported using pain relievers (non-steroidal anti-inflammatory drugs (NSAIDs), paracetamol etc., *p <* 0.0005). Finally, activities affected by severe pain were class attendance (*p* = 0.01), personal studying (*p* < 0.0005), exercising (*p* < 0.0005), and socializing (*p* < 0.0005). Exam attendance (*p* = 0.27) and clinical placement attendance (*p* = 0.48) were not affected by severe dysmenorrhea. Dysmenorrhea has a high prevalence among nursing students and seems to affect important aspects of wellbeing and academic performance when the pain intensity is severe. The present findings lay the foundation for further investigation of dysmenorrhea both in the Greek population and cross-culturally.

## 1. Introduction

Dysmenorrhea is one of the most common medical conditions and complaints voiced by women during their reproductive life. It is also a leading cause of absenteeism from school or college and work [1,2]. The Greek term of dysmenorrhea is globally used meaning “painful monthly bleeding”. This gynecological condition, affecting as many as 50% of menstruating women, usually takes place after 6–24 months of menarche. [3,4]. A wide variety of symptoms during menstruation have been noted, including lower abdominal pain, cramps, nausea, vomiting, headache, diarrhea [2], as well as fatigue, irritability [5] and depressive mood [6].

Dysmenorrhea can occur as primary dysmenorrhea or secondary dysmenorrhea. Primary dysmenorrhea is characterized by pain during menstruation without organic lesion in pelvis, caused by increased endometrial prostaglandin production [7,8]. Secondary dysmenorrhea is defined as painful menses associated with medical gynecological disorders such as endometriosis [9], adhesions, cysts, pelvic tumors, etc. [8].

Prevalence of primary dysmenorrhea varies from 45 to 95 percent globally [8]. Although dysmenorrhea is a very common condition in adolescence, it seems to be continued later in women’s life. However, primary dysmenorrhea tends to decrease with age [2], especially after 30 [10]. Pain severity and its consequences force women to use pharmacological and non-pharmacological treatments such as herbs, yoga, acupuncture, and aromatherapy [11]. Drugs mainly used are the non-steroidal anti-inflammatory drugs (NSAIDs) (first-line treatment), mefenamic acid, or oral contraception [12,13]. Their effectiveness is attributed to their action on prostaglandin as NSAIDs inhibit prostaglandin synthetase whereas oral contraceptives reduce endometrial thickness which is followed by a reduction of prostaglandin release [8,14]

Some studies have evaluated health behavior factors, such as eating habits, smoking, physical activity, or lifestyle, which have been shown to be predictors of the painful menses e.g., [15,16,17].

It has been shown that dysmenorrhea, either primary or secondary, affects women’s daily living, influencing their productivity (e.g., work, studies, socialization etc.) [18,19,20,21]. Severe pain, which is commonly accompanied by anxiety, depressed mood, and irritability, may result in female students’ decreased academic performance and social dysfunction, and can have a profound impact upon wellbeing, thereby impairing a woman’s quality of life [22].

Although dysmenorrhea is a condition which is globally examined, there are no recent studies that have investigated this common female medical condition in Greece. Therefore, the aim of the present study was to assess the prevalence of dysmenorrhea among university nursing students in Greece, to describe symptoms and pain characteristics and to evaluate impact of dysmenorrhea on students’ wellbeing concerning academic performance, physical exercise, and social functioning.

## 2. Materials and Methods

### 2.1. Study Design, Sampling and Setting

We conducted a descriptive cross-sectional study at the Nursing School Undergraduate Program of a university in Athens between May and October 2017. Female participants were recruited through convenience sampling among undergraduate nursing students during the academic semester. The inclusion criteria were a good knowledge of the Greek language and consent to participate in the study. Students that were on menopause or reported having a menstrual cycle duration of over 50 days were excluded from the analysis as oligomenorrhea might be indicative of transition to menopause (over 60 days, perimenopause) [23] or attributed to other underlying gynecological and endocrinological health conditions [24,25] Participants were informed on dysmenorrhea and basic menstrual terminology prior to completing the questionnaire.

### 2.2. Instrument

We designed and used a questionnaire of 40 items, to assess and determine the characteristics of dysmenorrhea. The questionnaire consisted of 3 parts. The first part included questions regarding demographic characteristics (e.g., living situation, age, height, weight, age of year studied), health behaviors (nutrition, exercising, smoking) and menstruation characteristics (menarche age, menstrual cycle duration). The second part focused on the characteristics of menstrual pain including questions on its appearance, onset, intensity, areas of pain, other symptoms, mood during menstruation (asking them whether they had normal mood, depressed mood, irritability, or tendency to fight) and use of pain relievers. Pain intensity was assessed using a Visual Analogue Scale (VAS) which ranged from 0 cm (no pain at all) to 10 (worst possible pain). Scores were categorized as such: 1–3.9 mild; 4–7.9 moderate; 8–10 severe. Finally, the third part included questions on the impact of dysmenorrhea on nursing students’ academic life. The questionnaire was prepared by the authors of the study and was reviewed by an expert in gynecology who was not part of the research team.

### 2.3. Ethical Approvals

The present study was approved by the general committee of the university nursing department. Prior to participating, participants were informed on the aims of the study, assured of the confidentiality of their data, and given verbal consent according to the principles provided by the Declaration of Helsinki.

### 2.4. Data Analysis

The participants’ body mass indexes (BMIs) were calculated using the reported weight and height values and categorized as such: <18.5 kg/m^2^: underweight 18.5–24.9 kg/m^2^: normal weight, 25.0–29.9 kg/m^2^: overweight and ≥30.0 kg/m^2^: obese. To simplify analysis and meet the chi-squared test assumption of equal or more than 5 expected counts, we recoded the variables on the functioning impact as such: “never” and “a few times” were categorized as “less affected”, and “quite often” and “always” were categorized as “more affected”. Descriptive statistics (frequencies, percentages, and means) were used to assess prevalence of dysmenorrhea, demographic and clinical characteristics, menstrual pain, and menstruation characteristics, mean age and mean age of menarche. Categorical variables were presented as *n*(%) and quantitative variables as mean ± standard deviation. Associations between dysmenorrhea status and health and clinical characteristics and dysmenorrhea intensity and impact on academic life (categorical variables) were assessed using Pearson’s χ^2^ when the assumption of the chi-squared test (≥20% of the cells with less than 5 expected counts) was met and Fisher’s exact test when this assumption was violated. To further analyze the significant effects, we used the z test with a Bonferroni correction. The effect of dysmenorrhea intensity on the number of days being absent from school and on the number of pain relievers used were analyzed using Kruskal–Wallis tests. A value of *p* < 0.05 was considered statistically significant. To enter and analyze the data, we used the IBM SPSS Statistics for Windows Version 25.0 (IBM Corp., Armonk, NY, USA).

## 3. Results

### 3.1. Students’ Demographic, Clinical and Menstrual Characteristics

Out of the 859 female students studying nursing, a total of 637 women (74.16%) agreed to participate in the study. However, 6 of them reported having a menstrual cycle duration of over 50 days so they were excluded from the analysis yielding several 631 participants. Table 1 shows the demographic and clinical characteristics of the sample. The mean age of the participants was 23.88 ± 7.34 years (range, 18–55 years). Most of our sample was within the 21–29 age group (43.5%) and the 18–20 age group (41.5%) whereas only 7.1% and 7.6% were aged between 30–39 and over 40 respectively. The vast majority were not married (85.7%), lived alone (22.3%), with their parents (62.4%) or co-habited with their partner (4.3%) whereas only 9.8% were married. 63.2% of them were from Athens whereas only 36.7% were from other places in Greece or abroad. Most of the sample reported having a Greek nationality (89.5%). Regarding the financial situation, 15.5% of the students reported a yearly family income of less than 5000 € whereas most students reported a yearly family income of less than 10,000 € (43.6%). Moreover, a relatively high rate of participants were first year students (37.2%) whereas only 6% were in their fourth year of study and 14.9% reported being students for more than four years.

Regarding health, 32.2% of the participants reported smoking, 54% of them reported exercising regularly and 59.2% reported following a healthy diet. The mean BMI was 22.66 ± 4.33 kg/m^2^ (range, 14.51–44.46). 65% of the students had a normal weight whereas 14.8% and 6.6% were overweight and obese, respectively.

Finally, regarding menstruation characteristics, the mean menarche age was 12.74 ± 1.57 years, the mean duration of menstruation was 5.85 ± 1.23 and the average menstrual cycle duration was 28.76 ± 3.4 days.

### 3.2. Prevalence and Characteristics of Dysmennorhea.

The prevalence of menstrual pain experience in the present sample was 89.2% (563 out of 631 women). Table 2 shows the pain intensity and dysmenorrhea characteristics in the subsample that reported having menstrual pain.

The prevalence of dysmenorrhea severity across the clinical and menstrual characteristics was also assessed (Table 3). There were no significant differences on smoking, exercising, BMI and Menstrual Cycle duration across mild pain, moderate pain, severe pain, and no pain groups (*p* > 0.1). There was a marginal effect of duration of menstruation on dysmenorrhea status (*p* = 0.051). *Z* tests with Bonferroni correction revealed no significant differences among the groups. However, it revealed a significant difference between the < 7 days and 7 ≥ days of menstruation on the severe pain group (*p* < 0.05). The count of the 7 ≥days subgroup was higher than the expected count (112 > 100.1 participants, *z* = 2.1) whereas the count of the < 7 days subgroup was lower than the expected count (179 < 190.9 participants, *z* = −2.1) suggesting a possible association of menstruation duration over 7 days and pain severity.

There were significant effects of healthy nutrition, family history of dysmenorrhea, menarche age, and participants’ age (*p* < 0.05). *Z* tests with Bonferroni correction on healthy nutrition showed no significant differences among groups on nutrition. Post-hoc *Z* tests on family history revealed that the severe group tended to report family history of dysmenorrhea more frequently (count: 73 > expected count: 58.6, *z* =2.9) than the no pain group (count: 7 < expected count: 14, *z* = −2.2) (*p* < 0.05) but no other differences among groups were noticed. Post-hoc comparisons on menarche age revealed that in the subgroup that reported experiencing their first menstruation at 13–15 years, there was a statistically significant difference between the severe pain and no pain groups (*p* < 0.05) in which the count of the severe group was lower than the expected count (104 < 117.3 participants, *z* = −2.2) whereas the count of the no pain group was higher than the expected one (36 > 27.3 participants, *z* = 2.3). No other differences across dysmenorrhea groups were noticed. However, regarding the differences across the menarche age subgroups, in the severe pain group, there was a statistically significant difference between the ≤12 age and the 13–15 age subgroups, where the count of the first subgroup was higher than the expected count (156 > 138.5 participants, *z* = 2.8) and the count of the second subgroup was lower than expected (104 < 117.3 participants, *z* = −2.2) showing that more participants of the severe group reported experiencing their first menstruation at age younger than 13 years old than experiencing at older ages. Finally, multiple comparisons on the age effect revealed that in the over 40 years subgroup, the severe group differed significantly with a lower frequency than the expected one (count: 13 participants < expected count: 20.9 participants, *z* = −2.4) from the no pain group (count: 12 participants > expected count: 4.6 participants, *z* = 3.7) (*p* < 0.05). Moreover, in the no pain group, the over 40 years subgroup had a higher count than the expected (count: 12 participants > expected count: 4.6 participants, *z* = 3.7) relatively to what was observed in both the 18–20 years (count: 23 participants < expected count: 25.3 participants, *z* = −0.6) and the 21–29 age subgroups (count: 20 participants < expected count: 26.7 participants, *z* = −1.8) (*p* <.05). This analysis suggests that in the present sample women over 40 years old experience less frequently severe menstrual pain than women younger than 30 years old. No other differences were yielded regarding participants’ age.

Characteristics of pain across dysmenorrhea status groups are shown in Table 4. There was no significant association between the Pain Onset and pain intensity (*p* = 0.43). However, there were significant differences between the groups on pain duration, the use of pain relievers and the number of pain relievers received (*p* < 0.05). Post-hoc comparisons in the pain duration revealed that for the less than 1 day pain duration subgroup, the mild pain group had a significantly higher percentage (69.9%, count: 55 > expected count: 28.5, *z* = 6.7) than both the moderate pain (35.8%, count: 68 = expected count: 65.9, *z* = 0.2) and the severe pain groups (25.8%, count: 75 < expected count: 102.6, *z* = −4.9) (*p* < 0.05). For the 1–3 days pain duration, the mild pain group showed a significantly smaller percentage (29.6%, count: 24 < expected count: 46.9, *z* = −5.6) than both the moderate pain (57.2%, count: 107 = expected count: 108.4, *z* = −0.3) and the severe pain groups (66.3%%, count: 193 > expected count: 168.7, *z* = 4.2) (*p* < 0.05). Moreover, in the mild pain group, significantly more participants reported a pain duration of less than 1 day than a duration of 1–3 days or more than 4 days (*p* < 0.05). In the severe pain group, women reported significantly more frequently than expected a pain duration of 1–3 days and over 4 days (6.9%, count: 20 > expected count: 14.1, *z* = 2.3) relatively to reporting a less than 1 day of pain duration (*p* < 0.05). This showed that women with moderate and severe pain tend to report a longer pain duration than women with mild menstrual pain.

Multiple comparisons on pain relievers use, showed that a significantly higher percentage of participants in the severe pain group reported using pain relievers (86.3%, count: 252 > expected count: 208.6, *z* = 8.1) than in the mild pain (36.6%, count: 30 < expected count: 58.6, *z* = −7.6) and the moderate pain groups (63.4%, count: 118 < expected count: 132.9, *z* = −3.0). Similarly, the mild pain group reported a significantly less frequent pain relievers use than the moderate pain group (*p* < 0.05). Post-hoc *p* correcting testing on the number of different pain relievers used during menstruation also revealed than the severe pain group reported using more different pain relievers in combination than the mild pain (*z* = −7.68, *p* < 0.0005) and moderate pain groups (*z* = −4.49, *p* < 0.0005). The moderate pain group also reported receiving more such drugs than the mild pain group (*z* = −4.07, *p* < 0.0005).

Finally, there was a significant association between time periods during menstruation with no pain and dysmenorrhea intensity (*p* < 0.0005). Post-hoc testing revealed that significantly more women with severe pain reported never experiencing a time period with no pain (35.6%, count: 104 > expected count: 75.9, *z* = 5.4) than women with either mild (12%, count: 10 < expected count: 21.6, *z* = −3.1) or moderate menstrual pain (17.1%, count: 32 <expected count: 48.6, *z* = −3.4). More women with moderate pain reported experiencing a pain free time period for a few times (72.7%, count: 136 > expected count: 116.5, *z* = 3.6) than women with mild (55.4%, count: 46 < expected count: 51.7, *z* = −1.4) and severe pain (57.5%, count: 168 > expected count: 181.9, *z* = −2.4). However, significantly more women with mild pain reported having pain free time periods quite frequently (30.1%, count: 25 > expected count: 8.9, *z* = 6.2) than women with either moderate (10.2%, count: 19 > expected count: 29, *z* = −0.3) or severe pain (5.5%, count: 16 > expected count: 31.2, *z* = −4.1).

### 3.3. Dysmenorrhea Intensity and Impact on Social Functioning, Exercising and Nursing Academic Life

Table 5 shows the impact of dysmenorrhea on wellbeing (exercising, socializing) and academic functioning). No significant differences between dysmenorrhea groups were noticed on exam absenteeism and clinical placement absenteeism (*p* > 0.1). However, there was a marginal effect of dysmenorrhea on failing a module due to menstrual pain (*p* = 0.08). *Z* tests with Bonferroni correction revealed no significant differences among the groups. However, it revealed a significant difference between the less affected and more affected subgroups on the severe pain group where the count of the more affected subgroup was higher than the expected count (18 > 12.9 participants, *z* = 2.1) whereas the count of the less affected subgroup was lower than the expected count (266 < 271.1 participants, *z* = −2.1) suggesting a possible impact of severe pain on exam performance.

However, there were significant effects of dysmenorrhea intensity on absenteeism from school, Days being absent from school, postponing personal studying, postponing exercising, and refraining from socializing (*p* < 0.05). Multiple comparisons on absenteeism showed that menstrual pain affected attending school more in the severe pain group (count: 35 participants > expected count: 25 participants, *z* = 3) than in the moderate pain group (count: 10 participants < 15.9 participants, *z* = −1.9). Post-hoc testing on the duration of absenteeism showed that severe pain group reported being absent from school more days than both the moderate pain group (*z* = −4.3, *p* < 0.0005) and the mild pain group (*z*= −5.41, *p* < 0.0005). Multiple comparisons on personal studying variable revealed that more women with severe pain reported stopping studying due to menstrual pain (27.3%, count: 78 participants > expected count: 52.1 participants, *z* = 5.7) than women with either mild (9.6%, count: 8 participants < expected count: 15.1 participants, *z* = −2.2) or moderate pain (8.1%, count: 15 participants < expected count: 33.7 participants, *z* = −4.4). Similarly, regarding exercising, the severe pain group reported significantly more frequently postponing physical exercising (50.9%, count: 144 participants > expected count: 116 participants, *z* = 4.9) than both the mild pain (23.2%, count: 19 participants < expected count: 33.6 participants, *z* = −3.6) and the moderate pain groups (33.7%, count: 62 < expected count: 75.4 participants, *z* = −2.5). Finally, the severe pain group also reported impact of dysmenorrhea on socializing significantly more frequently (29%, count: 82 participants > expected count: 50.4 participants, *z* = 7) than the mild pain (3.7%, count: 3 participants < expected count: 14.6 participants, *z* = −3.6) and the moderate pain groups (7%, count: 13 participants < expected count: 33 participants, *z* = −4.7).

## 4. Discussion

The aim of the present study was trifold. Firstly, we aimed to assess the prevalence of dysmenorrhea in a sample of nursing students in Greece. Secondly, we assessed the association between various demographic and clinical factors and dysmenorrhea. We also aimed to analyze the characteristics of menstrual pain. Finally, we investigated the academic performance, socialization, and exercising during menstruation in students with dysmenorrhea who reported different levels of pain severity. The prevalence of dysmenorrhea (defined as the appearance of pain while menstruating) in the present study was high (89.2%) as most students reported experiencing it. To the best of our knowledge, this is the first study measuring the prevalence of dysmenorrhea in women residing in Greece. A wide range of dysmenorrhea prevalence across different countries has been reported previously ranging between 16–91% [2]. The most common pain intensity reported was the severe one (52.5%) while only 13.6% students reported experiencing mild pain. This finding differs from what was reported previously in which the prevalence of severe intensity was much lower e.g., [2,19,26]. This discrepancy could be explained by the difference in pain perception across different cultures.

The lower abdomen was reported as the most common pain region in our sample (61.2%) accompanied by other menstrual symptoms such as swollen breast (24.1%), swollen abdomen (21.6%) and headache (13.3%). Regarding mood during menstruation, almost half of the participants with dysmenorrhea reported experiencing anger during menstruation (43.8%) whereas 24.8% reported feeling depressed. These symptoms were similar to what was reported previously e.g., [20,27,28]. Given that the present study and the previous ones that reported mood during menstruation have not administered valid questionnaires to assess it while women were menstruating, no safe inferences on mood during menstruation can be made. To the best of our knowledge, there are no studies that have assessed the association between mood and dysmenorrhea using valid tools of depression, anxiety, or anger while participants are menstruating and experiencing the pain. Future research could administer such valid tools to women with and without dysmenorrhea during menstruation and to compare their scores to those during the other menstrual cycle phases.

The effect of various clinical and menstrual characteristics that have been linked with dysmenorrhea previously was also investigated in the present study. We did not find differences in dysmenorrhea status (pain intensity and no pain) depended on smoking, exercising, BMI and menstrual cycle frequency. Previous research have shown a higher risk for dysmenorrhea in women with lower BMI, smoking behavior and a longer cycle but a lower risk in women that exercise regularly [10]. However, there are also studies that did not find differences in dysmenorrhea in relation to these characteristics e.g., [19,29]. This discrepancy across literature might be due to the different ages of the participants, cultures, sample size, definition of dysmenorrhea or scale measuring pain intensity. Moreover, in our study we did not measure the number of cigarettes smoked each day or the number of hours or intensity of exercising which may have affected these results. This discrepancy remains to be further investigated.

Nevertheless, we found associations between family history of dysmenorrhea, menarche age, duration of menstruation, participants’ age, and dysmenorrhea status. An association between family history and dysmenorrhea has also been shown previously e.g., [2,15,19,29,30] which might attributed to the learned reaction and perception of pain (e.g., pain catastrophizing [31]) from mothers to daughters or to genetic factors [32]. However, the significant effect concerned only the severe pain group and not the mild or the moderate intensity. Regarding menarche, we found that more participants with severe pain reported having their first menstruation at age 12 or younger than these that reported having it at older age. There was no association in the other dysmenorrhea groups. However, findings on menarche in previous research are contradictory. A systematic review on 29 studies had shown an increased risk for dysmenorrhea in women with early menarche (<12) [10]. On the other hand, other studies did not find such risk [2,19,28]. The reason for this difference across studies is not yet clear and there might be other factors that mediate this association. In our sample, more women over 40 years old reported experiencing no menstrual pain than women under 30 years old. Indeed it has been shown that younger age (<30) has an increased risk for dysmenorrhea [2,10]. A possible explanation for this finding could be that more women over 40 might have given birth than younger students. Livebirth had been shown to be associated with reduced prevalence of dysmenorrhea even after controlling for age [2,33]. However, we did not include a question on birth history so no safe suggestions on this can be made. No other differences were found among age groups and this could be explained by the low sample sizes in the 30–39 and ≥40 age groups.

We also showed that there were more participants with severe pain that they menstruate for over 7 days than expected suggesting a possible association between longer menstruation and severe pain. We did not find differences across the other pain severity groups. Previous findings in this association are contradictory. Previous studies showed that longer duration of menstrual flow (over 6–7 days) was risk factor for dysmenorrhea [15,34] and this has also been reported in a systematic review and meta-analysis of the literature on chronic pelvic pain [10]. However, other studies did not find such association e.g., [17,19]. The reason for this discrepancy in the literature is unknown but it could be explained by the differences in sample size across groups of dysmenorrhea status, menstrual flow duration of each sample (longer durations of over 7 days are less frequent in the population and therefore in the samples used) statistical power, definitions of dysmenorrhea and culture.

The use of pain relievers was also assessed in the present study. Most of the students with dysmenorrhea reported receiving pain relievers to manage their menstrual pain (71.4%). More participants with severe pain (86.3%) reported using pain relievers than participants with moderate (63.4%) and mild pain (36.6%). Participants with moderate pain also reported using pain relievers at higher rate than those with mild pain. High rates of pharmacologic pain relief has also been reported previously in Turkey e.g., [19,29], Brazil [21], Jordan [18], Mexico [26] etc. These findings suggest that there is a need in educating Greek students in responsible pain reliever use and in managing their menstrual pain with other non-pharmacologic ways of pain relief.

Regarding the impact of dysmenorrhea on functioning and wellbeing of the students during menstruation, we showed that severe pain negatively affected attendance of classes, personal studying, exercising, and socializing during menstruation. In all these variables, participants with severe pain significantly reported more negative impact than those with moderate or mild pain. Similar findings have also been observed in previous studies e.g., [18,19,20,21,26,29,34]. These findings suggest that dysmenorrhea is not a mere medical condition but it is also associated with mental health as it negatively affects quality of life during menstruation [22]. Indeed, dysmenorrhea has been shown to be associated with increased depression and anxiety as measured by self-reporting questionnaires [35,36].

Avoidance of exercise due to menstrual pain is counterproductive. Exercise has been shown to increase circulating endorphin levels [37] and have beneficial effects on depression [38] and acute exercise has been shown to decrease perception of experimentally induced pain [39]. Beneficial effects of regular exercise on menstrual pain has also been suggested by a recent systematic review on 15 Randomized Controlled Trials [40], though not implemented during menstruation. Given these, women with dysmenorrhea might benefit from regular exercise even during menstruation in both physical and psychological menstrual symptoms. However, this remains to be further investigated.

Refraining from socializing due to severe menstrual pain alongside psychological symptoms may contribute to the decreased quality of life during menstruation. Given that perceived social support has been shown to moderate the association between psychological symptoms and menstrual pain [41], decreased socialization and possibly disrupted social relationships during menstruation may also have a counterproductive effect in mood and pain during this menstrual phase in women with severe dysmenorrhea. However, analysis yielded an interesting finding. Only the situations that are easily avoided (lecture attendance that is not always compulsory in Greek Universities, studying, exercising, and socializing) were affected by severe dysmenorrhea. The situations that cannot be easily avoided were not affected (clinical placement and attendance in exams). The impact of dysmenorrhea on functioning could be mediated by psychological factors (e.g., pain catastrophizing [31], anxiety, depression or personality traits [42]). Future research could investigate whether the association between dysmenorrhea and impact on functioning is mediated by such factors.

As it was reported above, severe dysmenorrhea was associated with postponing personal studying. We also find a marginal effect of dysmenorrhea on exam performance in which more than expected students with severe dysmenorrhea reported having failed an exam due to the menstrual pain. Given that previously women with dysmenorrhea have reported experiencing decreased concentration e.g., while attending class due to their menstrual pain [19,43,44], it could be hypothesized that menstrual pain may affect attention. Future studies could test this hypothesis by examining the attention and other cognitive abilities of women with or without dysmenorrhea across the menstrual cycle by administering valid neuropsychological tests.

The present study was not free of limitations. Firstly, as it aimed to investigate the prevalence of dysmenorrhea generally in a sample of nursing students in Greece, there was no distinction between primary and secondary dysmenorrhea. Future studies in Greece could investigate the prevalence of these types of dysmenorrhea separately. Moreover, as the sample was limited to nursing students in an Athenian University, generalizability of the present results is probably limited. Given that students of health sciences might be more health conscious than other students, the present results might not generalize in women with no such training. Another limitation had to do with the cross-sectional nature of the present study. Given that pain intensity and impact in everyday activities might not be stable overtime and symptoms may improve across time [2], a future study in Greece employing a longitudinal design could provide a more clear view of this condition. Finally, the present study defined dysmenorrhea as the pain experienced during menstruation. However, the definition differs across the different studies as a standardized definition is not yet available. A future cross-cultural study could investigate the prevalence and characteristics of dysmenorrhea across different studies by using the same definition and same scale of pain intensity.

## 5. Conclusions

The present study showed a high prevalence of dysmenorrhea in a sample of women residing in Greece. It also showed some factors being associated with mostly severe dysmenorrhea, menstrual pain characteristics varying across the dysmenorrhea intensities, and impact of this condition in wellbeing (social function, exercising, and academic life). The present results highlight the need for implementing and testing the efficacy of pain management strategies in Greece and conducting further studies to better understand the prevalence, characteristics, correlates, and impact of dysmenorrhea in Greek women.

## Figures and Tables

**Table 1:** Demographic and Clinical Characteristics of the sample.

Characteristics	*n* (%)
Marital Status	
Married	62 (9.8%)
Not married	534 (84.6%)
Co-habiting with partner	27 (4.3%)
Did not report	8 (1.3%)
Origin	
Athens	398 (63.1%)
City or town with population of over 50,000	67 (10.6%)
Rural Area	165 (26.1%)
Did not report	1 (.2%)
Nationality	
Greek	565 (89.5%)
Other	66 (10.5%)
Yearly Family Income	
0–5000 €	98 (15.5%)
5000–1000 €	275 (43.6%)
10,000–20,000 €	163 (25.8%)
Over 2000 €	78 (97.3%)
Did not report	17 (2.7%)
Living Situation	
Living Alone	141 (22.3%)
With Parents	394 (62.4%)
With Housemate	80 (12.7%)
Did not report	16 (2.5%)
Year of Study	
1st year	235 (37.2%)
2nd year	140 (22.2%)
3rd year	115 (18.2%)
4th year	38 (6%)
After 4th year, upon completion	94 (14.9%)
Did not report	9 (1.4%)
Smoking	
Yes	203 (32.2%)
No	428 (67.8%)
Exercising	
Yes	340 (53.9%)
No	290 (46%)
Did not report	1 (0.2%)
Healthy Nutrition	
Yes	372 (59%)
No	256 (40.6%)
Did not report	3 (0.5%)
BMI	
Underweight (< 18.5 kg/m^2^)	80 (12.7%)
Normal Weight (18.5–24.9 kg/m^2^)	410 (65%)
Overweight (25–29.9 kg/m^2^)	92 (14.6%)
Obese (≥30.0 kg/m^2^)	41 (6.5%)
Did not report height and/or weight	8 (1.3%)
Height	1.66 ± 0.06 m ^1^
Weight	62.35 ± 12.85 ^1^

^1^ Mean ± standard deviation.

**Table 2:** Pain Intensity, region of pain, and other menstrual symptoms.

Characteristics	*n* (%)
Pain intensity	
Mild	74 (13.6%)
Moderate	185 (33.9%)
Severe	286 (52.5%)
Region of pain ^1^	
Lower abdomen	449 (61.2%)
Inguinal Region	88 (12%)
Lumbar	172 (23.4%)
Thigh	25 (3.4%)
Other symptoms ^1^	530 (97.2%)
Headache	204 (13.3%)
Dizziness	161 (10.5%)
Fainting	77 (5.0%)
Sweating	94 (6.1%)
Vomiting	47 (3.1%)
Diarrhea	116 (7.6%)
Loss of Appetite	86 (5.6%)
Swollen Abdomen	331 (21.6%)
Swollen Feet	49 (3.2%)
Swollen Breast	370 (24.1%)
Mood during Menstruation ^1^	
Normal	145 (21.4%)
Depressed	168 (24.8%)
Anger	297 (43.8%)
Tendency to fight	68 (10%)
Pain Relievers	
Yes	400 (71.4%)
No	160 (28.6)
Drug Compounds ^2^	
Non-steroidal anti-inflammatory drugs	216 (48.3%)
Paracetamol	193(43.2%)
Paracetamol + codeine	2 (0.4%)
Paracetamol + Muscle Relaxant	1 (0.2%)
Spasmolytic	34 (7.6%)
Sumatriptan for Migraines	1 (0.2%)

^1^ As multiple responses were given, numbers do not total to 563 women. ^2^ As multiple responses were given, numbers do not total to 400 women.

**Table 3:** The association between dysmenorrhea status and clinical, menstrual characteristics.

Characteristics	Mild Pain (*n* = 83, 13.2%)	Moderate Pain (*n* = 188, 29.8%)	Severe Pain (*n* = 292, 46.3%)	No Pain (*n* = 68, 10.8%)	Χ^2^(df), *p*
Smoking					2.12(3), *p* = 0.55
Yes	26 (31.3%)	54 (28.7%)	102 (34.9%)	21 (30.9%)	
No	57 (68.7%)	134 (71.3%)	190 (65.1%)	47 (69.1%)	
Exercising					3.03(3), *p* = 0.39
Yes	50 (60.2%)	94 (50.3%)	156 (53.4%)	40 (58.8%)	
No	33 (39.8%)	93 (49.7%)	136 (46.6%)	31.3 (41.2%)	
Healthy nutrition					8.42(3), *p* = 0.04
Yes	55 (67.1%)	122 (64.9%)	161 (55.3%)	34 (50.7%)	
No	27 (32.9%)	66 (35.1%)	130 (44.7%)	33 (49.3%)	
Family History of dysmenorrhea					10.30(3), *p* = 0.02
Yes	16 (19.3%)	32 (17.1%)	73 (25.6%) ^b^	7 (10.3%) ^a^	
No	67 (80.7%)	155 (82.9%)	212 (74.4%)	61 (89.7%)	
BMI					5.17(9), *p* = 0.82
Underweight	9 (10.8%)	28(15.2%)	33 (11.3%)	10 (15.4%)	
Normal Weight	59 (71.1%)	115 (62.5%)	194 (66.7%)	42 (65.6%)	
Overweight	10 (12.0%)	28 (15.2%)	47 (16.2%)	7 (10.8%)	
Obese	5 (6.0%)	13 (7.1%)	17 (5.8%)	6 (9.2%)	
Menarche Age					12.60(6), *p* = 0.05
≤12 years	35(42.2%)	85(45.2%)	156 (54.2%) ^13–15 years^	25 (37.3%)	
13–15 years	34 (41.0%)	81 (43.1%)	104 (36.1%) ^b^	36 (53.7%) ^a^	
>15 years	14 (16.9%)	22 (11.7%)	28 (9.7%)	6 (9.0%)	
Duration of menstruation					7.79(3), *p* = 0.051
<7 days	57 (69.5%)	132 (70.2%)	179 (61.5%)	52 (76.5%)	
≥7 days	25 (30.5%)	56 (29.8%)	112 (39.5%) ^<7 days^	16 (23.5%)	
Menstrual cycle					2.06, *p* = 0.97 ^1^
<21 days	1 (1.3%)	3 (1.8%)	2 (0.8%)	1 (1.8%)	
21–35	73 (96.1%)	156 (95.7%)	257 (96.6%)	55 (96.5%)	
>35	2 (2.6%)	4 (2.5%)	7 (2.6%)	1 (1.8%)	
Age					19.92(9), *p* = 0.02
18–20 years	32 (41.6%)	69 (38.5%)	121 (44.2%)	23 (37.7%) ^≥40^	
21–29 years	36 (46.8%)	81 (45.3%)	120 (44.5%)	20 (32.8%) ^≥40^	
30–39 years	3 (3.9%)	15 (8.4%)	18 (6.6%)	6 (9.8%)	
≥40 years	6 (7.8%)	14 (7.8%)	13 (4.7%) ^b^	12 (19.7%) ^a^	

^a^: Statistically significant different from severe pain, ^b^: Statistically significant different from no pain; ^1^ Fisher’s exact test.

**Table 4:** Dysmenorrhea intensity and pain characteristics.

Pain Characteristics	Mild Pain (*n* = 83, 13.2%)	Moderate Pain (*n* = 188, 29.8%)	Severe Pain (*n* = 292, 46.3%)	Χ^2^(df), or H, *p*
Pain Onset				3.86(4), *p* = 0.43
On the same day of menstrual flow	58 (73.4%)	119 (63.6%)	10 (12.7%)	
One day prior to menstrual flow	11 (13.9%)	36 (19.3%)	32 (17.1%)	
More than 2 days before menstrual flow	10 (12.7%)	32 (17.1%)	56 (19.4%)	
Pain Duration				53.50, *p* < 0.0005 ^1^
Less than 1 day	55 (69.9%) ^1–3d, b,c^	67 (35.8%)	75 (25.8%) ^1.3d, >4d^	
1–3 days	24 (29.6%) ^<1 day, b,c^	107 (57.2%)	193 (66.3%) ^<1^	
More than 4 days	1 (1.2%) ^<1 day^	6 (3.2%)	20 (6.9%) ^<1, uncertain^	
Uncertain	1 (1.2%)	7 (3.7%)	3 (1%) ^>4 d^	
Time periods with no pain				60.36, *p* < 0.0005 ^1^
Never	10 (12%)	32 (17.1%)	104 (35.6%) ^a, b^	
A few times	46 (55.4%)	136 (72.7%) ^never, a,c^	168 (75.5%) ^never, more than a few times, b^	
More than a few times	25 (30.1%) ^never, a few times, b,c^	19 (10.2%) ^a^	16 (5.5%) ^never, a few times, a^	
Always	2 (2.4%)	0 (0%)	4 (1.4%)	
Pain Relievers				86.25(2), *p* < 0.0005
Yes	30 (36.6%) ^b,c^	118 (63.4%) ^a,c^	252 (86.3%) ^a,b^	
No	52 (63.4%) ^yes^	68 (36.6%) ^yes^	40 (13.7%) ^yes^	
Number of pain relievers (0–4)	0 (1), 184.94 ^b,c^	1 (1), 262.06 ^a,c^	1 (0), 322.42 ^b,c^	H(2) ^2^ = 64.43, *p* < 0.0005

^a^: Statistically significant different from mild pain, ^b^: Statistically significant different from moderate pain, ^c^: Statistically significant different from severe pain; ^1^ Fisher’s exact test; ^2^ Kruskal–Wallis test; Data presented as either N (%) or Median (Interquartile Range), Mean Rank.

**Table 5:** Impact of Dysmenorrhea on functioning and wellbeing across pain intensity levels.

Pain Characteristics	Mild Pain (*n* = 83, 13.2%)	Moderate Pain (*n* = 188, 29.8%)	Severe Pain (*n* = 292, 46.3%)	Χ^2^(df), or H, *p*
Absenteeism from school				9.29(2), *p* = 0.01
Less affected	79 (96.3%)	176 (94.6%) ^c^	257 (88%) ^b^	
More affected	3 (3.7%)	10 (5.4%)	35 (12%) ^less affected^	
Days being absent from school	0 (2), 128.85	2 (3), 173.92 ^c^	3 (4), 227.06 ^a,b^	H(2) ^2^ = 38.41, *p* < 0.0005
Absenteeism on exams				2.06, *p* = 0.27 ^1^
Less affected	81 (98.8%)	185 (100%)	284 (99.3%)	
More affected	1 (1.2%)	0 (0%)	2 (0.7%)	
Clinical Placement Absenteeism				1.45(2), *p* = 0.48
Less affected	82 (98.8%)	179 (96.8%)	273 (96.1%)	
More affected	1 (1.2%)	6 (3.2%)	11 (3.9%)	
Postpone personal studying				32.51(2), *p* < 0.0005
Less affected	75 (90.4%)	170 (91.9%)	208 (72.7%) ^a,b^	
More affected	8 (9.6%) ^less affected^	15 (8.1%) ^less affected^	78 (27.3%) ^less affected^	
Failing a module due to dysmenorrhea				4.98(2), *p* = 0.08
Less affected	82 (98.8%)	179 (96.8%)	266 (93.7%)	
More affected	1 (1.2%)	6 (3.2%)	18 (6.3%) ^less affected^	
Postpone Exercising				26.27(2), *p* < 0.0005
Less affected	63 (76.8%)	122 (66.3%)	139 (49.1%) ^a,b^	
More affected	19 (23.2%) ^less affected^	62 (33.7%) ^less affected^	144 (50.9%) ^less affected^	
Refrain from Socializing				50.00(2), *p* < 0.0005
Less affected	79 (96.3%)	172 (93%)	201 (71%) ^a,b^	
More affected	3 (3.7%) ^less affected^	13 (7%) ^less affected^	82 (29%) ^less affected^	

^a^: Statistically significant different from mild pain, ^b^: Statistically significant different from moderate pain, ^c^: Statistically significant different from severe pain; ^1^ Fisher’s exact test; ^2^ Kruskal–Wallis test; Data presented as either N(%) or Median (Interquartile Range), Mean Rank.
